# Studies on [5,6]-Fused Bicyclic Scaffolds Derivatives as Potent Dual B-Raf^V600E^/KDR Inhibitors Using Docking and 3D-QSAR Approaches

**DOI:** 10.3390/ijms161024451

**Published:** 2015-10-15

**Authors:** Hai-Chun Liu, San-Zhi Tang, Shuai Lu, Ting Ran, Jian Wang, Yan-Min Zhang, An-Yang Xu, Tao Lu, Ya-Dong Chen

**Affiliations:** 1 School of Science, China Pharmaceutical University, Nanjing 211169, China; E-Mails: tuna888@126.com (H.-C.L.); tangsanzhi@163.com (S.-Z.T.); luai071@163.com (S.L.); tran1021@126.com (T.R.); wjnj12@163.com (J.W.); yanminzhang_cpu@126.com (Y.-M.Z.); fanqie0401@163.com (A.-Y.X.); 2 State Key Laboratory of Natural Medicines, China Pharmaceutical University, Nanjing 211169, China

**Keywords:** B-Raf/KDR, [5,6]-fused bicyclic scaffolds, docking, 3D-QSAR

## Abstract

Research and development of multi-target inhibitors has attracted increasing attention as anticancer therapeutics. B-Raf^V600E^ synergistically works with vascular endothelial growth factor receptor 2 (KDR) to promote the occurrence and progression of cancers, and the development of dual-target drugs simultaneously against these two kinds of kinase may offer a better treatment advantage. In this paper, docking and three-dimensional quantitative structure activity relationship (3D-QSAR) studies were performed on a series of dual B-Raf/KDR inhibitors with a novel hinge-binding group, [5,6]-fused bicyclic scaffold. Docking studies revealed optimal binding conformations of these compounds interacting with both B-Raf and KDR. Based on these conformations, comparative molecular field analysis (CoMFA) and comparative molecular similarity indices analysis (CoMSIA) 3D-QSAR models were constructed, and the best CoMFA (*q*^2^ = 0.542, *r*^2^ = 0.989 for B-Raf; *q*^2^ = 0.768, *r*^2^ = 0.991 for KDR) and CoMSIA models (*q*^2^ = 0.519, *r*^2^ = 0.992 for B-Raf; *q*^2^ = 0.849, *r*^2^ = 0.993 for KDR) were generated. Further external validations confirmed their predictability, yielding satisfactory correlation coefficients (*r*^2^_pred_ = 0.764 (CoMFA), *r*^2^_pred_ = 0.841 (CoMSIA) for B-Raf, *r*^2^_pred_ = 0.912 (CoMFA), *r*^2^_pred_ = 0.846 (CoMSIA) for KDR, respectively). Through graphical analysis and comparison on docking results and 3D-QSAR contour maps, key amino acids that affect the ligand-receptor interactions were identified and structural features influencing the activities were discussed. New potent derivatives were designed, and subjected to preliminary pharmacological evaluation. The study may offer useful references for the modification and development of novel dual B-Raf/KDR inhibitors.

## 1. Introduction

Over the past decades, the targeted therapy, which aims to develop highly selective inhibitors against a specific drug target, has become the primary paradigm in drug discovery [1,2,3]. However, many complex diseases, such as cancers, are not modulated by a single target, but rather an intricate network of signal pathways. Given the complexity of cancers, there is a general agreement that a multi-target approach might be more effective [4,5,6]. Therefore, drugs simultaneously modulating multiple targets have been extensively explored, which may help solve problems of single-target agents, such as limited efficacy, poor safety, and resistance. One of the multi-target strategies involves developing a single drug that can simultaneously act on two or multiple targets, which is more predictable on the pharmacokinetics and pharmacodynamics [7,8,9,10,11,12]. To date, more and more multi-target drugs have been successfully developed [7].

Members of the Raf family play a key role in the mitogen-activated protein kinase (MAPK) pathway. This pathway is closely related to cellular proliferation, differentiation, and survival [13]. In many cancers, oncogenic mutations of B-Raf are frequently observed and cause abnormal proliferation receptor tyrosine kinases (RTKs) without any control [14]. In addition, many solid tumors require sufficient amount of nourishment and new blood vessels are constructed for their progression in the aid of vascular endothelial growth factor (VEGF), which stimulates adjacent vascular endothelial growth factor receptor 2 (VEGFR-2 or KDR). The VEGFR-2 plays a significant role in the progression of solid tumors [15]. B-Raf with VEGFR-2 works synergistically to promote certain cancers, and to develop multi-target drugs simultaneously against these two kinases may provide a better therapeutic advantage. Thus, dual B-Raf and VEGFR-2 inhibitors could be effective therapeutic agents for cancer treatment [16]. Fortunately, the high degree of sequence and structural homology among the ATP-binding pockets of B-Raf and VEGFR-2 makes it plausible to design dual B-Raf and VEGFR-2 inhibitors [17,18]. Sorafenib is the first kinase drug that targets both Raf/Mek/Erk and VEGFR-2/PDGFR signaling cascades to block the tumor cell proliferation and inhibit the tumor angiogenesis [19]. Sorafenib is a type II inhibitor that interacts with the inactive DFG-out conformations of wild type B-Raf and oncogenic mutant B-Raf^V600E^ as well as VEGFR-2 [20,21,22]. Studies have indicated that Type II inhibitors have certain advantages compared to Type I inhibitors, which includes the improvement of biochemical efficiency and selectivity [23]. Thus, dual inhibition of B-Raf and VEGFR-2 may provide strong antitumor efficacy, especially Type II inhibitors.

To design multi-target agents, theoretical studies on the structural features of the compounds may provide useful information. Molecular docking methodology is robust in the study of protein-ligand interaction, and quantitative structure-activity relationship (QSAR) methodology is considered to be the most effective in understanding the structure and activity relationship and designing new chemical entities [24,25,26]. The three-dimensional quantitative structure activity relationship (3D-QSAR) studies, including comparative molecular field analysis (CoMFA) and comparative molecular similarity indices analysis (CoMSIA) [27,28], help to identify the interaction fields surrounding the compounds according to their corresponding activity, which may shed light on designing of novel B-Raf and KDR inhibitors and help with the issue of commonality and selectivity among B-Raf and KDR members. Therefore, combined docking and 3D-QSAR studies [29,30,31,32,33] may offer useful information for designing and obtaining more potent new dual kinase inhibitors.

**Figure 1:**
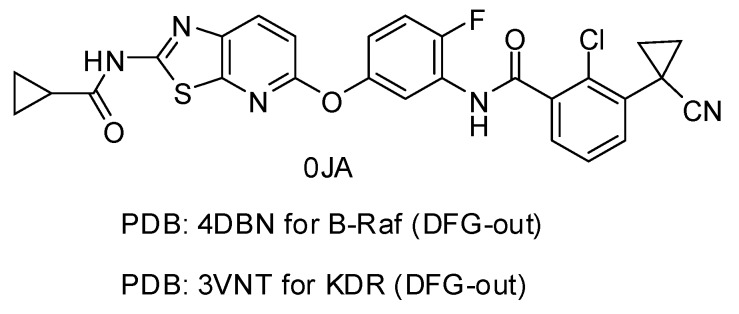
Chemical structure of 0JA as B-Raf/KDR inhibitor. PDB: protein data bank; KDR: vascular endothelial growth factor receptor 2.

Recently, molecules with a novel hinge-binding group have been reported to show dual inhibition against both B-Raf and KDR, which form two hydrogen bond interactions with hinge region Cys amino acid residues through various [5,6]-fused bicyclic scaffolds. These derivatives were proven to be the highly potent DFG-out type RAF/VEGFR-2 inhibitors. The representative compound 0JA (Ligand from the cocrystal structure of VEGFR-2 (PDB code: 3VNT) and B-Raf (PDB code: 4DBN)) showed potent inhibitory activity against B-Raf^V600E^ (IC_50_ = 7.0 nM) and wild-type B-Raf (12 nM), C-Raf (1.5 nM) as well as VEGFR-2 (2.2 nM) (Figure 1) [34,35,36,37]. In this paper, novel series of [5,6]-fused bicyclic derivatives as dual B-Raf and KDR inhibitors were carried out in a theoretical study using the docking and 3D-QSAR methods. The binding modes and conformations of these dual-target inhibitors with both B-Raf and KDR were determined by docking, and 3D-QSAR models were established based on docked conformations. The model is expected to facilitate deeper understanding of the structure activity relationship of these compounds, and provide useful information for rational design of novel and potent dual B-Raf and KDR inhibitors.

## 2. Results and Discussion

### 2.1. Structure Alignment Analyses

**Figure 2:**
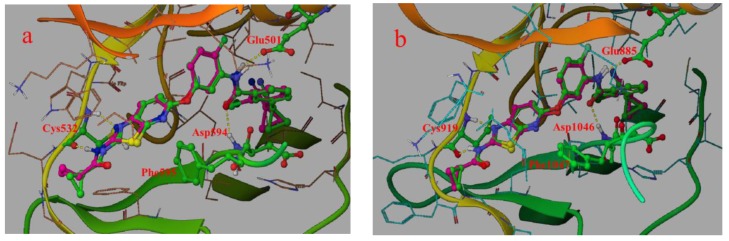
Overlay of the docked pose (green) of ligand 0JA with its co-crystal conformation (pink): (**a**) B-Raf and (**b**) KDR.

The accuracy and reliability of the 3D-QSAR model is dependent on two important factors: the biological conformation selection and the structural alignment rule. In this study, the 3D-QSAR model is constructed by the docking-based conformation alignment. To ensure molecular docking could recapture feasible conformation, Glide-SP docking algorithm was first validated by re-docking of the crystal ligand. The best docking pose of compound 0JA (good docking score was observed) was only minimally differed from the co-crystal ligand conformation with a root mean-square deviation (RMSD) of 0.4967 and 0.2836 Å (Table 1, Figure 2a,b), suggesting high accuracy and reliability of Glide in reproducing the experimental binding mode.

**Table 1:** Docking and RMSD values for conformation validated.

PDB Code	Resolution (Å)	Ligand Code	IC_50_ (nM)	G_Score	RMSD (Å)
4DBN	3.15	0JA	7	−13.04	0.4967
3VNT	1.64	0JA	2.2	−14.69	0.2836

RMSD: root mean-square deviation; PDB: protein data bank.

Ligand 0JA showed significant binding interactions with the key residue of the target protein in the docking study. 0JA acted on the DFG-out conformation of B-Raf (PDB code: 4DBN). The [1,3]-thiazolo-[5,4-b]-pyridine-2-amine moiety formed the hydrogen bonds with the carbonyl and NH of Cys532 in the hinge region. The core was positioned in a hydrophobic cleft consisting of Ile463, Val471, Ala481, Trp531, Phe583 and Phe595. The central phenyl occupied the hydrophobic pocket adjacent to the ATP binding site. Carbonyl and NH of the biphenyl amide moiety formed hydrogen bonds with Asp594 and Glu501, respectively. Terminal phenyl extended into a back hydrophobic pocket generated by the Phe595 movement and lined with Val504, Leu505, Ile513, Ile572 and His574.

Docking of ligand 0JA in the X-ray co-crystal structure of KDR (PDB code: 3VNT) was depicted in Figure 2b. Similar to B-Raf, 0JA acted on the DFG-out conformation of KDR. The [1,3]-thiazolo-[5,4-b]-pyridine core was positioned in a hydrophobic cycle lined with Leu840, Ala866, Phe918 and Leu1035. Similar hydrogen bond interactions were observed with the carbonyl and NH of Cys919 in the hinge region. The central phenyl also occupied the hydrophobic pocket adjacent to the ATP binding site. Carbonyl and NH of amide formed hydrogen bonds with Asp1046 and Glu885, respectively. The terminal phenyl extended into a back hydrophobic pocket created through the Phe1047 flip and lined with Ile868, Leu889, Ile892, Leu1019 and His1026.

**Figure 3:**
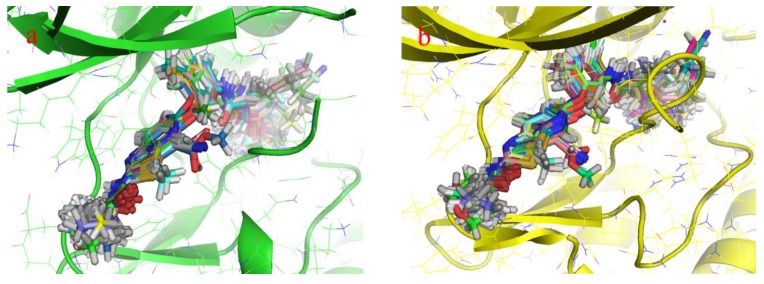
Molecular alignments of 40 inhibitors obtained from the docked conformation-based alignment: (**a**) B-Raf and (**b**) KDR.

To obtain accurate binding conformations, all compounds (Table 2) were docked into the active site of B-Raf and KDR kinases by using the Glide-SP docking algorithm. Figure 3a,b show the 3D models of molecular alignments of 40 compounds at the binding site of B-Raf and KDR. Docking conformations of all studied compounds were similar to co-crystal ligand 0JA conformation, providing an excellent alignment. Taken together, results from the Glide docking based alignments were suggested to be reasonable.

**Table 2:** Chemical structures and bioactivity of the training set and test set compounds.

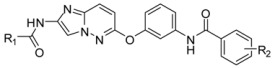								
Compound	R_1_			R_2_			Kinase IC_50_ (nM)	
							B-Raf^V600E^	VEGFR2
1-1 *	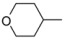			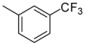			43	3.1
1-2	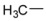			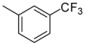			4.1	1.1
1-3	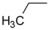			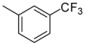			6.9	1.7
1-4	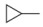			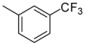			7.6	1.9
1-5	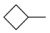			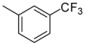			28	4.8
1-6	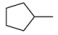			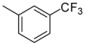			44	9.3
1-7	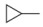			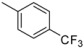			81	1.9
1-8	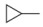			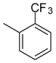			3700	1.4
1-9	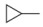			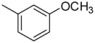			18	0.31
1-10	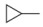			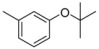			15	1.8
1-11	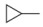			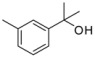			10	0.82
1-12	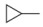			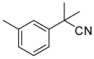			14	1.5
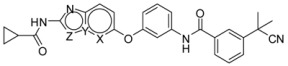								
**Compound**	**Ring**						**Kinase IC_50_ (nM)**	
							**B-Raf^V600E^**	**VEGFR2**
2-1	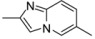						49	1.8
2-2	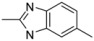						41	8.1
2-3	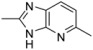						17	3.1
2-4	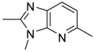						200	2.4
2-5 *	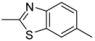						25	14
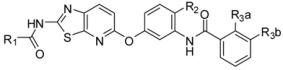								
**Compound**	**R_1_**		**R_2_**		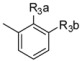		**Kinase IC_50_ (nM)**	
							**B-Raf^V6^°°^E^**	**VEGFR2**
3-1	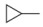		H		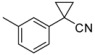		3.0	2.2
3-2	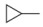		H		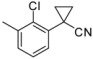		9.0	4.0
3-3 *	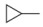		F		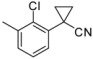		7.0	2.2
3-4	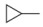		Cl		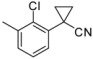		43	7.5
3-5	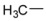		F		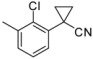		6.3	3.4
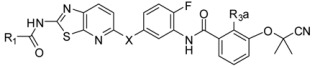								
**Compound**	**R_1_**		**X**		**R_3a_**		**Kinase IC_50_ (nM)**	
							**B-Raf^V600E^**	**VEGFR2**
3-6	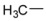		N-Me		H		23	10
3-7	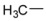		N-Me		Cl		45	14
3-8 *	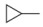		N-Me		Cl		38	7.5
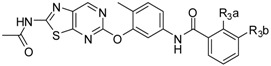								
**Compound**	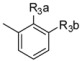						**Kinase IC_50_ (nM)**	
							**B-Raf^V600E^**	**VEGFR2**
4-1	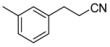						51	410
4-2	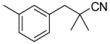						100	300
4-3	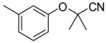						73	510
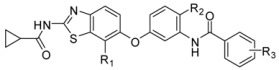								
**Compound**	**R_1_**		**R_2_**		**R_3_**		**Kinase IC_50_ (nM)**	
							**B-Raf^V600E^**	**VEGFR2**
5-1	NO_2_		H		*m*-C(CH_3_)_2_CN		25	70
5-2	CN		H		*m*-C(CH_3_)_2_CN		13	76
5-3 *	CO_2_Me		H		*m*-C(CH_3_)_2_CN		12	33
5-4	CH_2_OH		H		*m*-C(CH_3_)_2_CN		24	7.4
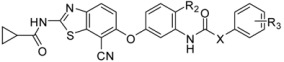								
**Compound**	**X**		**R_2_**		**R_3_**		**Kinase IC_50_ (nM)**	
							**B-Raf^V600E^**	**VEGFR2**
6-1 *	NH		H		*o*-CF_3_		19	120
6-2 *	NH		H		*p*-CF_3_		18	330
6-3	NH		F		*m*-CF_3_		49	730
6-4	>NH		>F		>*p*-CF_3_		>26	>660
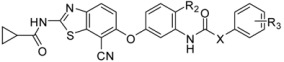								
**Compound**	**X**		**R_2_**		**R_3_**		**Kinase IC_50_ (nM)**	
							**B-Raf^V600E^**	**VEGFR2**
6-5	CH_2_		H		*o*-CF_3_		14	100
6-6	CH_2_		H		*m*-CF_3_		1.3	150
6-7	CH_2_		H		*p*-CF_3_		9.5	190
6-8	CH_2_		F		*m*-CF_3_		2.4	160

VEGFR2: vascular endothelial growth factor receptor 2; * Compounds in the test set.

### 2.2. 3D-QSAR Analyses

The CoMFA and CoMSIA models for B-Raf and KDR were performed based on docking conformations alignment. The statistical parameters of PLS analyses and external validation results were listed in Table 3.

**Table 3:** Summary of PLS and external validation results of CoMFA and CoMSIA (SEHA) analyses.

Statistical Parameters	B-Raf		KDR	
	CoMFA	CoMSIA	CoMFA	CoMSIA
*q*^2^	0.542	0.519	0.768	0.849
N	6	9	6	6
*r*^2^	0.989	0.992	0.991	0.993
SEE	0.075	0.068	0.107	0.089
*F*-value	376.623	299.397	453.385	649.012
*r*^2^_pred_	0.764	0.841	0.912	0.846
Field contributions				
Steric	0.457	0.179	0.486	0.107
Electrostatic	0.543	0.356	0.514	0.334
Hydrophobic		0.256		0.271
Hydrogen Bond Donor				
Hydrogen Bond Acceptor		0.210		0.288

PLS: partial least squares; CoMFA: comparative molecular field analysis; CoMSIA: comparative molecular similarity analysis; *q*^2^: Leave-one-out (LOO) cross-validated correlation coefficient; N: optimum number of components; *r*^2^: non cross-validated correlation coefficient; SEE: standard error of estimate; *F*-value: the Fischer ratio; *r*^2^_pred_: predicted correlation coefficient for test set compounds; S, E, H, D, A: steric, electrostatic, hydrophobic, as well as hydrogen bond donor and acceptor fields, respectively.

#### 2.2.1. 3D-QSAR Results for B-Raf Kinase

**Figure 4:**
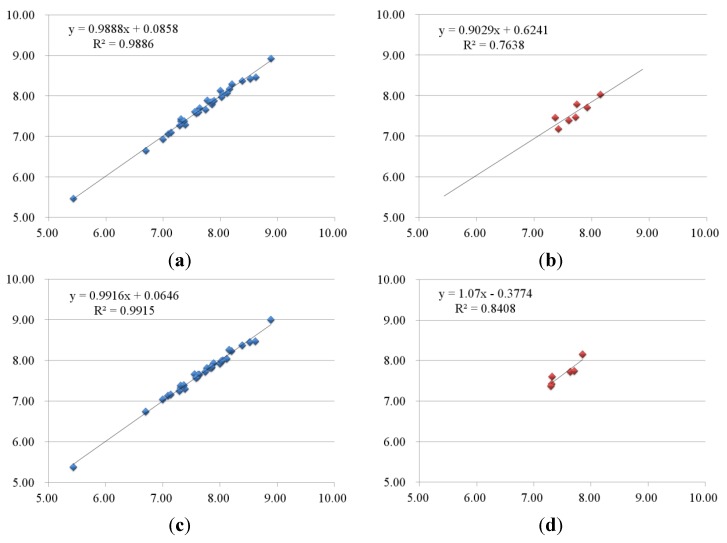
Plots of predicted activities *vs.* actual ones for (**a**,**b**) CoMFA and (**c**,**d**) CoMSIA analyses, in which 33 compounds in the training set were expressed as blue rectangles and seven compounds in the test set were expressed as red rectangles.

Through PLS statistical analysis, Glide docking based CoMFA model for B-Raf kinase showed a reliable cross-validated correlation coefficient *q*^2^ of 0.542 and optimal number of components of 6, indicating a good internal prediction of model. The CoMFA model also exhibited a high correlation coefficient *r*^2^ of 0.989 with relatively lower SEE of 0.075 and relatively higher *F* value of 376.623 in the final non-cross-validated model. The contribution of steric fields and electrostatic fields was 45.7% and 54.3%, respectively, which indicated that both steric field and electrostatic field had equally important influences. The above values suggested a good statistical correlation and a good internal predictive ability of the CoMFA model as shown in Figure 4a.

The optimal CoMSIA model was explored by using different combinations of steric (S), electrostatic (E), hydrophobic (H), hydrogen bond donor (D), and acceptor (A) fields. To get a clear view, only models whose *q*^2^ exceeds 0.5 were considered. The final optimal CoMSIA (SEHA) model gave significant results with *r*^2^ of 0.992, *q*^2^ of 0.519, *F* of 299.397, and SEE of 0.068. The contributions of steric, electrostatic, hydrophobic, and hydrogen bond acceptor fields are 17.9%, 35.6%, 25.6%, and 21.0%, respectively. Figure 4c depicted the relationship between the actual and predicted pIC_50_ values for the optimal CoMSIA model. The above statistical values suggested that a satisfactory CoMSIA model was obtained.

In order to further validate the models’ predictive ability, activities of test set compounds not included in the construction of the 3D-QSAR models were predicted (shown in Table 4). Both CoMFA and CoMSIA exhibited satisfactory results in term of predictive correlation coefficient *r*^2^_pred_ of 0.764 and 0.841, respectively. The favorable *r*^2^_pred_ values indicated good external predictive ability and were able to predict inhibitory activities of new inhibitors. The experimental, predicted and residuals of test set molecules were shown in Table 4 and the correlations between the actual and predicted pIC_50_ values of test set compounds are presented in Figure 4b,d. These statistical data and graphs indicated that the predicted values were in agreement with the experimental values in the allowable error range.

**Table 4:** The actual pIC_50_, predicted pIC_50_ and their residuals of the studied compounds for CoMFA and CoMSIA (SEHA) analyses.

No.	B-Raf					KDR				
	Actual pIC_50_	CoMFA		CoMSIA		Actual pIC_50_	CoMFA		CoMSIA	
		pIC_50_	Res.	pIC_50_	Res.		pIC_50_	Res.	pIC_50_	Res.
Training set										
1-2	8.39	8.38	0.01	8.37	0.02	8.96	8.76	0.2	8.89	0.07
1-3	8.16	8.18	−0.02	8.27	−0.11	8.77	8.84	−0.07	8.82	−0.05
1-4	8.12	8.07	0.05	8.04	0.08	8.72	8.90	−0.18	8.70	0.02
1-5	7.55	7.61	−0.06	7.66	−0.11	8.32	8.41	−0.09	8.33	−0.01
1-6	7.36	7.35	0.01	7.35	0.01	8.03	8.04	−0.01	8.12	−0.09
1-7	7.09	7.06	0.03	7.13	−0.04	8.72	8.83	−0.11	8.86	−0.14
1-8	5.43	5.47	−0.04	5.38	0.05	8.85	8.77	0.08	8.89	−0.04
1-9	7.74	7.66	0.08	7.73	0.01	9.51	9.53	−0.02	9.32	0.19
1-10	7.82	7.85	−0.03	7.82	0	8.74	8.78	−0.04	8.75	−0.01
1-11	8.00	8.14	−0.14	7.92	0.08	9.09	9.12	−0.03	9.08	0.01
1-12	7.85	7.80	0.05	7.82	0.03	8.82	8.87	−0.05	8.83	−0.01
2-1	7.31	7.39	−0.08	7.32	−0.01	8.74	8.66	0.08	8.75	−0.01
2-2	7.39	7.29	0.1	7.30	0.09	8.09	7.99	0.1	7.98	0.11
2-3	7.77	7.89	−0.12	7.81	−0.04	8.51	8.55	−0.04	8.50	0.01
2-4	6.70	6.65	0.05	6.74	−0.04	8.62	8.49	0.13	8.65	−0.03
3-1	8.52	8.42	0.1	8.45	0.07	8.66	8.42	0.24	8.48	0.18
3-2	8.05	8.03	0.02	8.00	0.05	8.40	8.47	−0.07	8.39	0.01
3-4	7.37	7.38	−0.01	7.39	−0.02	8.12	8.06	0.06	8.24	−0.12
3-5	8.20	8.29	−0.09	8.23	−0.03	8.47	8.52	−0.05	8.48	−0.01
3-6	7.64	7.70	−0.06	7.66	−0.02	8.00	7.98	0.02	7.95	0.05
3-7	7.35	7.36	−0.01	7.35	0	7.85	7.81	0.04	7.83	0.02
4-1	7.29	7.27	0.02	7.25	0.04	6.39	6.47	−0.08	6.44	−0.05
4-2	7.00	6.94	0.06	7.04	−0.04	6.52	6.55	−0.03	6.54	−0.02
4-3	7.14	7.11	0.03	7.16	−0.02	6.29	6.31	−0.02	6.31	−0.02
5-1	7.60	7.63	−0.03	7.60	0	7.15	7.28	−0.13	7.10	0.05
5-2	7.89	7.88	0.01	7.93	−0.04	7.12	7.19	−0.07	7.27	−0.15
5-4	7.62	7.61	0.01	7.65	−0.03	8.13	8.12	0.01	8.29	−0.16
6-3	7.31	7.43	−0.12	7.38	−0.07	6.14	6.30	−0.16	6.13	0.01
6-4	7.59	7.58	0.01	7.58	0.01	6.18	6.04	0.14	6.08	0.1
6-5	7.85	7.85	0	7.85	0	7.00	7.03	−0.03	6.97	0.03
6-6	8.89	8.93	−0.04	9.00	−0.11	6.82	6.79	0.03	6.88	−0.06
6-7	8.02	7.98	0.04	7.98	0.04	6.72	6.65	0.07	6.67	0.05
6-8	8.62	8.46	0.16	8.47	0.15	6.80	6.75	0.05	6.75	0.05
Test set										
1-1	7.37	7.46	−0.09	7.30	0.07	8.51	8.15	0.36	8.29	0.22
2-5	7.60	7.40	0.2	7.32	0.28	7.85	8.14	−0.29	7.83	0.02
3-3	8.15	8.03	0.12	7.85	0.3	8.66	8.41	0.25	8.29	0.37
3-8	7.42	7.18	0.24	7.31	0.11	8.12	7.92	0.2	7.83	0.29
Training set										
5-3	7.92	7.72	0.2	7.35	0.57	7.48	7.20	0.28	8.06	−0.58
6-1	7.72	7.47	0.25	7.64	0.08	6.92	7.06	−0.14	6.67	0.25
6-2	7.74	7.80	−0.06	7.71	0.03	6.48	6.33	0.15	6.14	0.34

Res.: residual values.

#### 2.2.2. 3D-QSAR Results for KDR Kinase

The 3D-QSAR analysis of KDR was performed on the same training set for B-Raf. The CoMFA model yielded *q*^2^ = 0.768 and *r*^2^ = 0.991, respectively. It was obvious that CoMSIA models also presented well in terms of higher *q*^2^ = 0.849 and *r*^2^ = 0.993 values. Other statistical parameters of PLS analyses were listed in Table 3.

**Figure 5:**
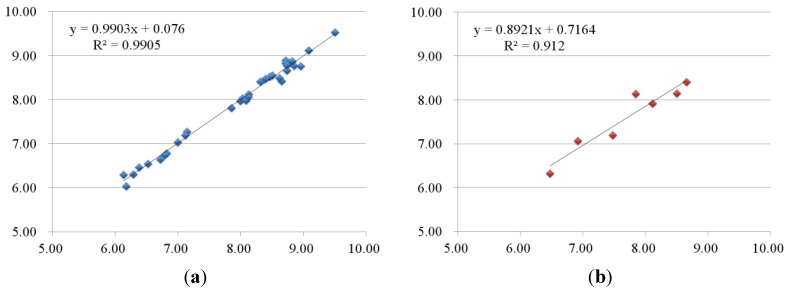

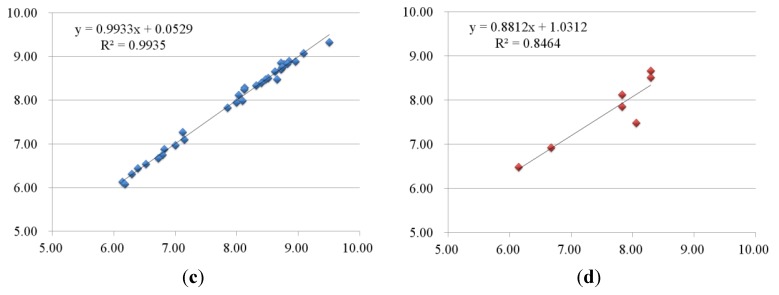
Plots of predicted activities *vs.* actual ones for (**a**,**b**) CoMFA and (**c**,**d**) CoMSIA analyses, in which 33 compounds in the training set were expressed as blue rectangles and seven compounds in the test set were expressed as red rectangles.

The actual activities (pIC_50_), the predicted activities and the corresponding residual values of the training set for the CoMFA and optimal CoMSIA models are listed in Table 4. Graphical representations of actual *vs.* predicted activities of training set are shown in Figure 5a,c. The CoMFA and optimal CoMSIA models possessed high *q*^2^ and *r*^2^ values, indicating that models had good internal predictive ability.

To validate the external predictability of the models, the *r*^2^_pred_ values were calculated for test set. As shown in Table 3 and Table 4, the *r*^2^_pred_ values of CoMFA and optimal CoMSIA models spanned from 0.912 to 0.846, revealing that models had good external predictive ability and could be used to predict the biological activities of novel compounds. The plots of actual *vs.* predicted activities of test set were shown in Figure 5b,d, showing that the predicted activities were in good agreement with the actual data.

### 2.3. Contour Maps

**Figure 6:**
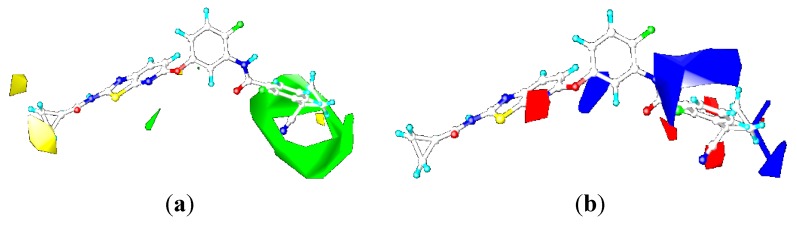
CoMFA contour maps of the ligand 0JA for B-Raf: (**a**) steric contour map and (**b**) electrostatic contour map.

**Figure 7:**
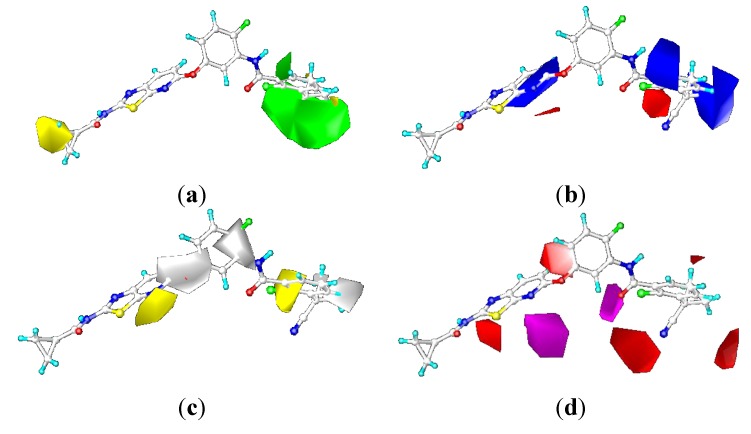
CoMSIA contour maps of the ligand OJA for B-Raf: (**a**) steric contour map; (**b**) electrostatic contour map; (**c**) hydrophobic contour map; and (**d**) hydrogen-bond acceptor contour map.

**Figure 8:**
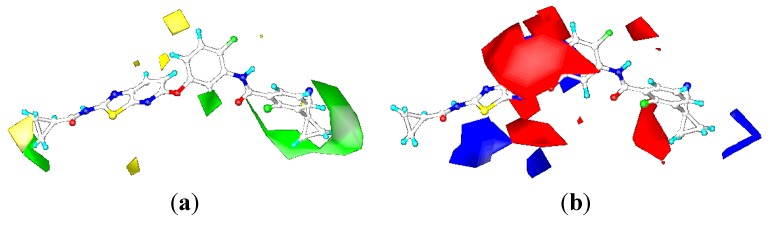
COMFA contour maps of the ligand 0JA for KDR: (**a**) steric contour map and (**b**) electrostatic contour map.

To visualize the results of the CoMFA and CoMSIA models more directly, the 3D coefficient contour maps of CoMFA (steric and electrostatic fields) and CoMSIA (steric, electrostatic, hydrophobic, and hydrogen bond acceptor fields) were generated (Figure 6, Figure 7, Figure 8 and Figure 10), respectively. To facilitate the analysis, ligand 0JA was selected as the reference in the 3D coefficient contour maps. The results of the CoMFA and CoMSIA models were graphically interpreted by the field contribution maps.

#### 2.3.1. Contour Maps for B-Raf

##### CoMFA Contour Maps

The contour maps of CoMFA (steric and electrostatic fields) are shown in Figure 6. In the contour map of steric field, green contour showed sterically favored region while yellow region indicated the area where bulky groups may cause decline in the inhibition activity of compounds. In the contour map of electrostatic field, red contour showed the region where electronegative group was favorable to increase the inhibitory activity while opposite was for blue contours.

In the contour map of steric field (Figure 6a), a large green contour was observed around the cyanocyclopropyl group of 2-chloro-3-(1-cyanocyclopropyl)benzene ring (ring-C), suggesting the bulky substituent was favored at this region such as methoxyl, trifluoromethoxyl, and cyanocyclopropyl. This bulky hydrophobic interaction may had an important role in enhancing the cellular activity against B-Raf, which was illustrated by the experimental fact that compound 1-10 exhibited higher activity than corresponding compound 1-9. The small green contours were found at the N atom position on the pyridine ring of [1,3]-thiazolo-[5,4-b]-pyridine scaffold (ring-A), around which the moderate-sized substituent was favored such as cyano. The cyclopropyl group near a hinge was surrounded by two yellow contours, which suggested bulky groups at this position would be unfavorable for binding the B-Raf protein. Because the position existed in a narrow space formed by the indole ring of Trp531 and Gly534, and that the optimal size for the narrow space would be smaller than the pyranyl group. This may explain why compound 1-1 with a bulky group showed significantly decreased activities than other compounds with a minor substituent near a hinge. Therefore, replacement of the pyranyl group in compound 1-1 with a methyl group in compound 1-2 showed significantly increased B-Raf inhibitory activity (IC_50_ = 4.1 nM). This was also well illustrated by the order of activity for these compounds: 1-4 > 1-5 > 1-6.

Electrostatic contour map was shown in Figure 6b. In general, red contours are close to heteroatoms, whose partial atomic charges were negative such as nitrogen and oxygen. The moderate red contours were found close to the N atom position on the pyridine ring of [1,3]-thiazolo-[5,4-b]-pyridine scaffold, indicating negative charged groups was favored. This trend can be reflected by the activities of compounds 5-2, 5-3, 6-5, 6-6 and 6-7 which all had the cyano group. These compounds were more active than corresponding compound 2-5. The 2-chloro-3-(1-cyanocyclopropyl)benzene ring was surrounded by three small red contours, suggesting that electronegative potential was preferred at these positions, such as F, Cl, OC(CH_3_)_2_CN, C(CH_3_)_2_OH, and CF_3_. The red contours above the phenyl of the benzene amide group demonstrated that the negative-favorable substituents induced the positive carbon of phenyl ring. With respect to the favorable positive potential, the blue region around the phenyl urea and benzene amide group showed key positive-favorable property, which reflected an important H-bond donor feature of NH group, consistent with the docking study. The blue contours were distributed around the central benzene ring (ring B) group, suggesting that positive charged groups increase the activity. This was consistent with the increase in the potency of compound 3-3 as compared to compound 3-2, due to the substitution of hydrogen atom with fluorine atom in the central phenyl group.

##### CoMSIA Contour Maps

The contour maps of CoMSIA steric and electrostatic fields are shown in Figure 7a,b, which were found to be quite similar to the contour maps derived from the above CoMFA model except several slight differences, and thus were not discussed here. The following analyses focus on the CoMSIA hydrophobic and hydrogen bond acceptor contour maps.

In hydrophobic contour map of CoMSIA, yellow and gray contours indicated hydrophobic and hydrophilic groups were favored, respectively. Figure 7c showed hydrophobic contour map of CoMSIA, gray contour was located around the linker between the ring-A and ring-B, which suggested hydrophilic contributions had effect on the activity. This may explain why compounds 3-6, 3-7 and 3-8 with an N-Me substituent were less active than compounds only with NH or O substituent here. The gray contour near the cyclopropyl position of the terminal phenyl group of compound 0JA indicated that hydrophilic substitution at this position was favorable. This may explain why compound 4-2 possessing a more hydrophobic substituent (–CH_2_C(CH_3_)_2_CN) showed significantly decreased activities than other compounds (4-1, 4-3) with a less hydrophobic substituent (e.g., –CH_2_CH_2_CN, –OC(CH_3_)_2_CN).

As indicated in Figure 7c, the white region was close to the polar CONH group and R-position of the ring-B, which interacted with hydrophilic amino acid residues, such as Glu501, Thr508, and Asp594. Hydrophobic substituents at R-position of the ring-B such as –F, –Cl were not beneficial to the improvement of inhibitory activity. For example, compounds 3-2, 6-2 and 6-6 with hydrogen at this position were more active than compounds 3-4, 6-4 and 6-8 with a Cl or F group at the same position. The yellow regions were situated around the ring-C. It was shown that the position points to the hydrophobic pocket of the binding site, interacting with the Ile513, His574, and Ile572 residues. It was shown that N1 of the ring-A is surrounded by the yellow region. This can be explained by the fact that compounds with more hydrophilic substitution (e.g., COOH) had decreased activities than compounds 5-3 and 5-4 with CO_2_Me and CH_2_OH as substituents.

The magenta contours indicate hydrogen bond-accepting groups increase the inhibitory activity, whereas the red contours indicate hydrogen bond-accepting groups decrease the activity. A magenta contour located on the carbonyl oxygen of the urea or NHCO moiety suggested that hydrogen bond-accepting groups were favored. This is obvious from the fact that the carbonyl oxygen participated in hydrogen-bonding interaction with the Asp594. The docking study also showed that the carbonyl forms a hydrogen-bond with Asp594. Another magenta contour was found near the N atom position on the pyridine ring of [1,3]-thiazolo-[5,4-b]-pyridine scaffold, which indicated that H-bond acceptor groups were favored there. Thus, it was not surprising that compounds 5-2 and 5-3 with –CN and –CO_2_Me as substituents had higher activities than corresponding compound 2-5. A red contour located on the S atom position of the thiazole pyridine ring of [1,3]-thiazolo-[5,4-b]-pyridine scaffold, indicating that H-bond acceptor groups were unfavorable there. The remaining red contour was around the H atom of the pyridine ring indicating that the hydrogen bond acceptor substituent was inessential for the increased inhibitory activity. Hence, compounds 4-1, 4-2, 4-3 did not show improved potencies than corresponding unsubstituted derivatives. Moreover, there were red acceptor-unfavorable contours over the 2-chloro-3-(1-cyanocyclopropyl)benzene ring of the template molecule, suggesting that the hydrogen bond acceptor group was not tolerated at this position. It was also in line with the above result that there was a hydrogen bond donor site near this region. For example, compound 1-11 with a –OH as substituent showed higher activity than corresponding compounds 1-10 and 1-12.

#### 2.3.2. Contour Maps for KDR

##### CoMFA Contour Maps

**Figure 9:**
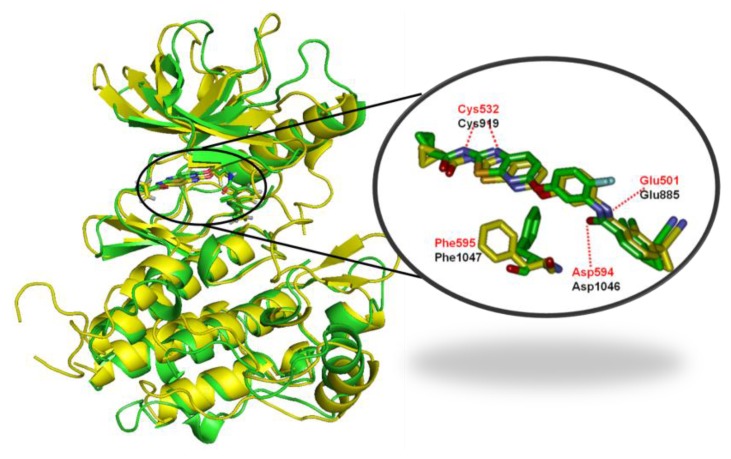
Structure alignment of B-Raf (4DBN, green) and KDR (3VNT, yellow) bound to inhibitor 0JA. (Cys532, Glu501, Asp594 and Phe595 are the key amino acids of B-Raf; Cys919, Glu885, Asp1046 and Phe1047 are the key amino acids of KDR).

The contour maps of the CoMFA steric and electrostatic fields for KDR are shown in the Figure 8a,b. They were similar to the contour maps of the CoMFA steric and electrostatic fields of B-Raf (Figure 6a,b). However, substitution of the N-acyl group (R1) had negligible impact on the KDR inhibition, with compounds 1-1, 1-2, 1-3, 1-4, 1-5, and 1-6 showing nano-molar order inhibitory activity against KDR. For example, in the steric contour map there was a small yellow contour beside N position at the C-7 position on the pyridine ring of [1,3]-thiazolo-[5,4-b]-pyridine scaffold, indicating that introduction of bulky groups here would decrease the KDR inhibitory activity. This was the opposite with steric contours of B-Raf. This can explain the favorable activity of compounds 2-5, and the unfavorable activity of compounds 5-1, 5-2, and 5-3. The reasons accounting for the aforementioned phenomenon may be as follows: there was a prominent difference in the Phe residue conformations of the DFG motif for B-Raf and KDR (Figure 9). The benzene ring of Phe595 in B-Raf (green) was located under the thiazolo-[5,4-b]-pyridine scaffold, whereas that of KDR (yellow) was located in front of the N atom position of pyridine. Consequently, the introduction of a bulky substituent could reduce the KDR inhibitory activity by steric repulsion. Studies found the C-7 position should be very important in modulating the ratio of KDR IC_50_ to B-Raf IC_50_. On the basis of the notable difference, we could design inhibitors to increase B-Raf selectivity over KDR.

In the electrostatic field of CoMFA model of KDR, there is a blue contour located on the S atom position of [1,3]-thiazolo-[5,4-b]-pyridine scaffold, indicating that positive charged groups increased the activity there. With respect to electrostatic contours of B-Raf, no blue contour located on the S position of thiazole, whereas there was blue contour around the amide linker group for B-Raf, which showed a key positive/favorable property and reflected an important H-bond donor feature of NH. According to the compound data, the NHCO substructure connected to ring-B and ring-C could apparently form two significant hydrogen bonds with Glu501 and Asp594. In order to study the structure-activity relationship, the linker between ring-B and ring-C was modified to explore appropriate linkers. An important modification for the linker was insertion of X (NH or CH_2_). The insertion of amine (X = NH) of ureides 6-1, 6-2, 6-3, 6-4 could form an additional hydrogen bond with Glu501, and the insertion of methylene (X = CH_2_) of the acetamides 6-5, 6-6, 6-7, and 6-8 was considered flexible enough so that ring-C could adjust the conformation to be accommodated into the hydrophobic back pocket of B-Raf. However, the impact of these chemical modifications of the linker for KDR was negligible.

##### CoMSIA Contour Maps

**Figure 10:**
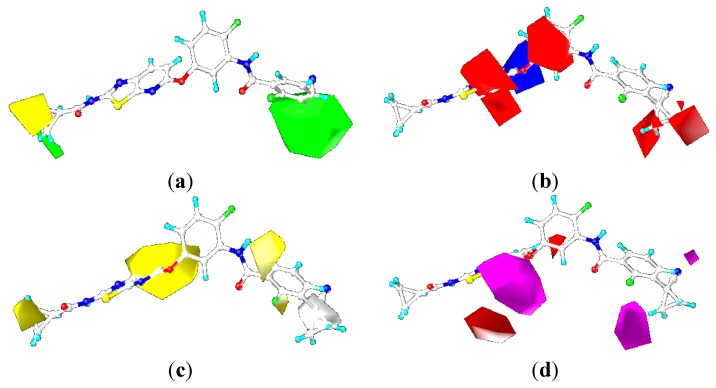
CoMSIA contour maps of the ligand OJA for KDR: (**a**) steric contour map; (**b**) electrostatic contour map; (**c**) hydrophobic contour map; and (**d**) hydrogen-bond acceptor contour map.

As shown in Figure 10, the CoMSIA steric and electrostatic contour maps (Figure 10a,b) were almost identical with CoMFA contour maps. Thus, the following discussion focuses on the hydrophobic and hydrogen bond acceptor fields. The contour maps of the hydrophobic and hydrogen bond acceptor fields of the CoMSIA model can be seen clearly in Figure 8c,d. They were also similar to the CoMSIA hydrophobic and hydrogen bond acceptor contour maps of B-Raf (Figure 7c,d).

The hydrophobic fields are presented in Figure 10c, yellow and white contours indicate hydrophobic and hydrophilic groups were favored, respectively. Compared with the hydrophobic contour of B-Raf, a yellow polyhedron that covered cyclopropane group near the hinge region suggested that a moderate-sized hydrophobic group could help increase the biological activity. This could be validated by the fact that the activity of compounds 1-2, 1-3, and 1-4 had better biological activity than compound 1-1. While no white contours are close to the central phenyl ring, indicating that hydrophilic substitution at this position was not necessary.

Figure 10d showed contour maps of CoMSIA hydrogen bond acceptor field. Compared to the hydrogen bond acceptor field of B-Raf, the magenta contour near *o*-Cl of the terminal phenyl ring-C revealed that hydrogen bond acceptor groups might be beneficial for the potency. The results were supported by the higher potency of compounds 1-8, 6-1, and 6-5 with CF_3_ at this position than corresponding compound 1-4 as well as compounds 1-7, 6-2, 6-6 and 6-7. Without magenta contour regions around urea or amide groups, the CO of urea or amide groups could provide one additional H-acceptor, which leads to a lower inhibitory activity.

### 2.4. Comparison for B-Raf and KDR Kinases

#### 2.4.1. Comparison of the Protein Structures

**Figure 11:**
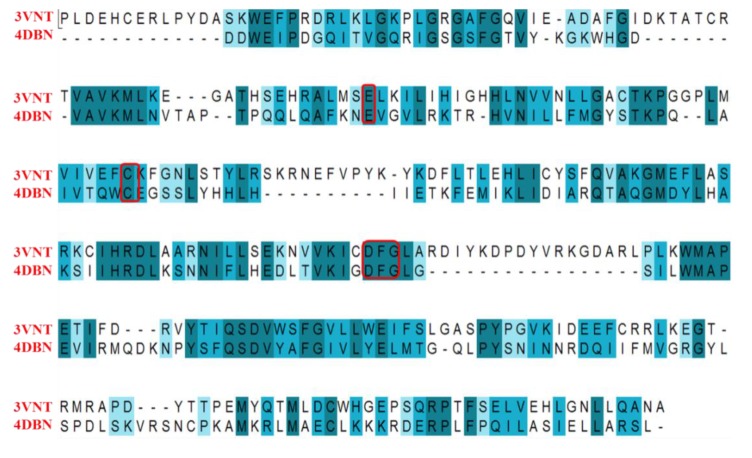
Sequence alignment of 4DBN (B-Raf) and 3VNT (KDR). Deep Blue color regions represent that the amino acid residues in the individual column were identical in the sequence alignment. The key amino acids in the binding sites were marked in red rectangles.

To compare these two proteins, the sequences were aligned and the crystal structures were superposed for B-Raf (PDB code: 4DBN) and KDR (PDB code: 3VNT). The comparison of the sequences and proteins are shown in Figure 9 and Figure 11.

The sequence identity and similarity were 25.0% and 46.2%, respectively. Identity and similarity of B-Raf and KDR chains were not very high, but B-Raf and KDR had a lot of common key points. As displayed in the alignment of the sequences, the same sequence ID for B-Raf and KDR was marked in blue shadows. The key amino acids in the active sites of B-Raf (PDB code: 4DBN) and KDR (PDB code: 3VNT) were marked in red rectangles. It can be observed that the docking active sites for B-Raf and KDR may have almost the same active sequence domains. Figure 9 depicted the structural superposition of B-Raf (PDB code: 4DBN) and KDR (PDB code: 3VNT), where the binding conformation and interaction were illustrated, with the root mean square deviation (RMSD) of 1.964 Å. The binding conformation and interaction of ligand 0JA with the two kinase receptors B-Raf and KDR were strikingly similar. Almost all key amino acids in the active sites (such as Glu501, Cys532, and Asp594 for B-Raf, and Glu885, Cys919, and Asp1046 for KDR) interacting with the [5,6]-fused bicyclic derivatives were well aligned for B-Raf and KDR, which may explain why these compounds are dual B-Raf /KDR inhibitors.

#### 2.4.2. Comparison of the 3D-QSAR Results

From the statistical results listed in Table 3, the CoMFA and CoMSIA models for B-Raf and KDR kinases exhibited excellent predictive powers. The statistical results of the CoMFA and CoMSIA models for B-Raf and KDR were very similar. For CoMFA models, both the steric and electrostatic fields both had equally important influences on the ligand-receptor interactions, with approximately 50% contributions to the steric and electrostatic fields, respectively. Furthermore, optimal CoMSIA models of B-Raf and KDR were constructed by using the same combination of steric, electrostatic, hydrophobic and hydrogen acceptor fields, indicating that the ligand binding to both B-Raf and KDR may be the similar. In addition, the electrostatic and hydrophobic fields were observed to play crucial roles for the interaction, with contributions of 61.2% (electrostatic: 35.6%, hydrophobic: 25.6%) and 60.5% (electrostatic: 33.4%, hydrophobic: 27.1%) in the CoMSIA models for B-Raf and KDR, respectively.

#### 2.4.3. Comparison of the 3D-QSAR Contour Maps

**Figure 12:**
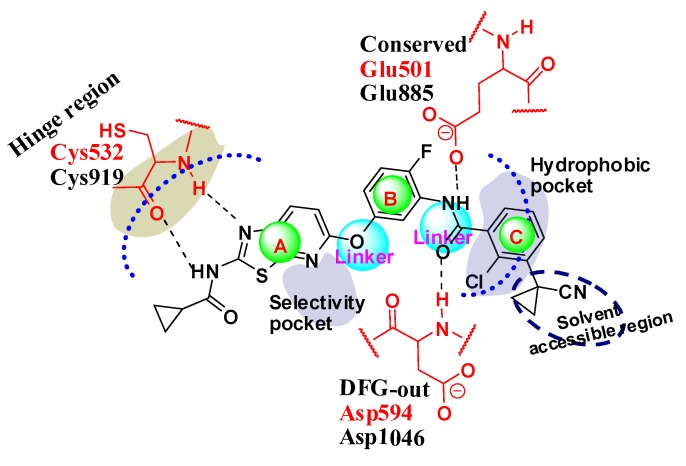
Structure activity relationship summarization for B-Raf and KDR dual inhibitors based on docking and 3D-QSAR investigation. The dotted line and the circle in dotted line represented near the region around the corresponding molecular groups.

Many similarities in sequences and protein structures of B-Raf and KDR may lead to very similar structural features of the B-Raf and KDR inhibitors. Through analysis and comparison of the B-Raf and KDR 3D-QSAR contour maps (Figure 6, Figure 7, Figure 8 and Figure 10), very similar structural requirements and small differences could be found for potent ligands of B-Raf and KDR kinases (Figure 12). From the discussion above, we could conclude common structural requirements for more potent dual inhibitors against B-Raf and KDR as follows:

(i) Ring-A and linker between ring-B and ring-C form key H-bonds with kinase.

(ii) Ring-A, -B, and -C are hydrophobic to interact with the Adenine site, DFG motif toward the allosteric site; and the cyanocyclopropyl-position should be hydrophilic-favorable.

However, some small differences between the contour maps of B-Raf and KDR could also be easily found. These differences may contribute to better understanding of their specific structural requirements for kinase selectivity.

### 2.5. Designing Potent Derivatives for Further Verification

Based on the results of established receptor-ligand interactions and 3D-QSAR above, and for further verification and design consideration, some novel compounds based on the 1H-benzo[d]imidazole moiety [38] with potential inhibitory activity were designed as B-Raf^V600E^/KDR dual inhibitors. The chemical structures and predicted activity values by the CoMFA and optimal CoMSIA models are shown in Table 5. The designed molecules were synthesized and the preliminary pharmacological evaluation would be used to verify the biological activity and our design strategy. The experimental inhibitory activities of designed new compounds were also listed in Table 5. The preliminary biological tests showed new compounds have dual B-Raf^V600E^/KDR inhibitory activity. Among them, compound D2 had the best inhibitory effect with the IC_50_ of 420 and 210 nM for B-Raf^V600E^ and KDR kinases, respectively. The results showed these theoretical results would provide the useful references and guidelines for optimizing current molecules and designing novel inhibitors with new scaffolds.

**Table 5:** The predicted pIC_50_ and B-Raf^V600E^ and KDR inhibitory activities of designed new compounds.

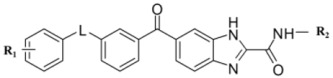											
No.	L	R_1_	R_2_	CoMFA		CoMSIA		IC_50_ (μM)		Actual pIC_50_	
				B-Raf	KDR	B-Raf	KDR	B-Raf^V600E^	KDR	B-Raf	KDR
D1	CONH	4-CH_3_	CH_3_	6.69	6.69	7.47	7.47	0.28	0.99	6.55	6.00
D2	CONH	3-Cl	CH_3_	7.14	7.14	7.83	7.83	0.42	0.21	6.38	6.68
D3	CONH	4-Cl	CH_3_	6.96	6.96	7.55	7.55	>5	0.95		6.02
D4	CONH	3-F	CH_3_	6.80	6.80	7.63	7.63	> 5	0.89		6.05
D5	CONH	3,4-diCl	CH_3_	7.23	7.23	7.7	7.7	0.15	>5	6.82	
D6	CONH	3-CF_3_	CH_2_CH_3_	7.49	7.49	7.94	7.94	>5	0.39		6.41
D7	NHCONH	3-CF_3_	CH(CH_3_)_2_	7.44	7.44	8.13	8.13	>5	1.52		5.82
D8	NHCONH	3-CF_3_	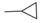	6.68	6.68	7.51	7.51	>5	1.32		5.88
Sorafenib								0.033	0.32		

## 3. Experimental Section

### 3.1. Data Set

For 3D-QSAR analyses, 40 compounds from the literature [34,35,36,37] were employed, which covered a range of more than three magnitudes (1.3–3700 nM for B-Raf, 0.31–730 nM for KDR) for their activity. The IC_50_ values of all compounds for B-Raf and KDR inhibition were converted to the −log IC_50_ (pIC_50_) as dependent variables in the 3D-QSAR analysis. The 3D structures for all 40 compounds were built and minimized, and then Gasteiger-Hückel charge was calculated. All works were all done in SYBYL 6.9 (Tripos, Inc.: St. Louis, MO, USA, 2002) [39].

Normally, the whole dataset for 3D-QSAR study would be divided into the training set and test set randomly. However, here, we performed an alternative way by a data mining methods of clustering [40,41], which may bring us more reliable results. In the clustering process, the dataset was clustered into 7 classes (shown in Table 2) according to the molecular descriptors and structural difference. The compounds were chosen from the above 7 categories with a ratio of about 4/1 of the training set and test set, according to biological activity and structural diversity. So 33 compounds as training set and the other 7 ones (asterisked in Table 2) as test set were selected to construct and validate the model, respectively.

### 3.2. Conformational Alignment

To construct reliable 3D-QSAR models, structure alignment is considered the most important and critical step [42]. The most common method is to align compounds onto a common scaffold. For our 3D-QSAR study, molecular docking based alignments were shown as the most effective alignment by fully considering the binding mode of compounds. Molecular docking can be used for alignment because it can dock compounds into the target binding site with similar modes. Meanwhile, the docking studies would also be conducive to the view of ligand-receptor interaction, assisting in further understanding the structure-activity relationship.

Glide 5.5 was selected to generate biological conformation as it has shown good reproducibility and accuracy in molecular docking and scoring [43,44]. The crystal structures of B-Raf and KDR (PDB code: 4DBN and 3VNT) were prepared with the Protein Preparation Wizard workflow in Schrödinger suite 2009 software (Schrödinger, LLC, New York, NY, USA, 2009). The grid files were generated, which were defined by the co-crystal ligand itself. All remaining parameters were kept as default. The compounds were flexibly docked into the binding site with standard precision (SP) docking mode. The optimal conformation of each compound was retained based on the Glide score and interaction modes.

### 3.3. 3D-QSAR Models

#### 3.3.1. Generation of 3D-QSAR Model

The CoMFA and CoMSIA analyses were carried out using the QSAR module in SYBYL6.9. In CoMFA study [27], the standard steric and electrostatic fields were calculated in the cubic lattice by a sp^3^ hybridized carbon with a +1.0 charge and grid spacing of 2.0 Å. The cut off value was set to 30 kcal/mol and the column filtering was set to be 2.0 kcal/mol for both fields. In CoMSIA study [28], steric, electrostatic, hydrophobic, and hydrogen bond donor and acceptor fields were calculated at each lattice by a sp^3^ carbon probe atom with a charge of +1.0 and a grid of 2 Å. The attenuation factor was set to the default value of 0.3.

#### 3.3.2. Partial Least Squares (PLS) Analysis and Validation

The Partial Least-Squares (PLS) [45,46] algorithm was employed for the structure-activity relationship. Biological activities (pIC_50_ values) as dependent variables and the field descriptors as independent variables were used to construct 3D-QSAR models. In cross validation, leave-one-out (LOO) algorithm was adopted to yield the optimal number of components (ONC), and cross-validation coefficient (*q*^2^), calculated with Equation (1):

(1) $$q^{2}=1-\frac{\sum {\left({\text{Y}}_{\text{pred}}-{\text{Y}}_{\text{exp}}\right)}^{2}}{\sum {\left({\text{Y}}_{\text{exp}}-{\text{Y}}_{\text{mean}}\right)}^{2}}$$

where Y_pred_, Y_exp_ and Y_mean_ are the predicted activity values, experimental activity values and mean activity values of training set compounds, respectively.

In the non-cross validation, the correlation coefficient (*r*^2^) was calculated using the obtained ONC. Additionally, the statistical significance of the models was also described by the SEE and F probability value. The predictive abilities of the obtained models were assessed by predicting the activities of the external test set composed of 7 compounds. The predictive correlation coefficient *r*^2^ (*r*^2^_pred_) reflected the predictive power of the CoMFA and CoMSIA models.

## 4. Conclusions

In this study, molecular docking and 3D-QSAR methods were synergistically applied to study the interaction of [5,6]-fused bicyclic derivatives as potent B-Raf/KDR inhibitors, and construct robust 3D-QSAR models for designing new dual B-Raf/KDR inhibitors. The optimal conformations of these compounds with both B-Raf and KDR kinases were revealed by docking studies. Based on the alignment of docking conformations, 3D-QSAR models showed satisfactory statistical quality and predictive ability with high *q*^2^ and *r*^2^ as well as low SEE. Furthermore, it was interesting to find good consistency of ligand-receptor interactions for B-Raf/KDR kinases from docking analyses and the 3D-QSAR studies. The filed distribution information of the 3D-QSAR contour maps played significant roles for the inhibitory activity against both B-Raf and KDR kinases. In addition, molecular docking along with 3D-QSAR contour maps offered detailed visual information to understand the intermolecular interaction between inhibitors and B-Raf/KDR. The key amino acids that affect the intermolecular interaction were identified. Some main structural factors that determine activities were discussed in detail. New potent derivatives were designed, synthesized and carried out preliminary biological evaluation. The results showed theoretical studies could offer useful information for designing novel potent dual B-Raf/KDR inhibitors.
